# RIPK1 expression and inhibition in tauopathies: implications for neuroinflammation and neuroprotection

**DOI:** 10.3389/fnins.2024.1530809

**Published:** 2025-01-27

**Authors:** Ignacio Silva-Llanes, Enrique Madruga, Ana Martínez, Isabel Lastres-Becker

**Affiliations:** ^1^Department of Biochemistry, School of Medicine, Universidad Autónoma de Madrid (UAM), Madrid, Spain; ^2^Instituto de Investigación Sanitaria La Paz (IdiPaz), Madrid, Spain; ^3^Instituto de Investigaciones Biomédicas “Sols-Morreale” UAM-CSIC, Madrid, Spain; ^4^Centro de Investigaciones Biológicas Margarita Salas (CSIC), Madrid, Spain; ^5^Centro de Investigación Biomédica en Red de Enfermedades Neurodegenerativas (CIBERNED), Madrid, Spain

**Keywords:** neuroinflammation, TAU, RIPK1, neurodegeneration, Alzheimer’s disease (AD), progressive supranuclear palsy (PSP), frontotemporal dementia (FTD)

## Abstract

Tauopathies are a group of neurodegenerative diseases characterized by the alteration/aggregation of TAU protein. One of the main challenges of these diseases is that they have neither biomarkers nor pharmacological targets to stop the neurodegenerative process. Apart from the neurodegenerative process, tauopathies are also characterized by a chronic low-grade neuroinflammation process, where the receptor-interacting protein kinase 1 (RIPK1) protein plays an essential role. Our research aimed to explore the role of RIPK1 in various tauopathies. We examined mouse models of frontotemporal dementia (FTD), as well as brain tissue samples from patients with progressive supranuclear palsy (PSP), a primary form of 4R tauopathy, and Alzheimer’s disease (AD), which is considered a secondary tauopathy. Our findings show elevated levels of *RIPK1* mRNA levels across various forms of tauopathies, in both mouse models and human tissue samples associated with primary and secondary TAU-related disorders. Furthermore, we investigated the potential of using a RIPK1 inhibitor, known as GSK2982772, in a mouse model as a novel treatment strategy for FTD. The data showed that GSK2982772 treatment effectively reduced the reactive astrocyte response triggered by TAU^P301L^ overexpression. However, this RIPK1 inhibitor failed to protect against the neurodegeneration caused by elevated TAU^P301L^ levels in the hippocampal region. These results suggest that although inhibiting RIPK1 activity may help reduce TAU-related astrogliosis in the brain, the complexity of the inflammatory pathways involved could explain the absence of neuroprotective effects against TAU-induced neurodegeneration.

## 1 Introduction

The protein TAU forms the main component of thread-like structures found inside cells, which are hallmarks of various brain disorders collectively known as tauopathies. These conditions include Alzheimer’s disease (AD), frontotemporal lobar degeneration (FTLD-TAU), progressive supranuclear palsy (PSP), and corticobasal degeneration, among others (Arendt et al., 2016; Zhang et al., 2022; Brackhan et al., 2023). Inherited mutations in the *MAPT* gene, which encodes TAU, are linked to familial frontotemporal dementia (FTD) (Hasegawa et al., 1998; Mirra et al., 1999; Rizzu et al., 1999; van Swieten et al., 1999). Examples include TAU^P301L^ and TAU^P301S^. The proline residue at codon 301 is part of the highly evolutionarily conserved PGGG motif, present in all microtubule-binding repeats, and is specifically located within the microtubule-binding repeat unique to 4R TAU (Rizzu et al., 2000). Such TAU mutations diminish the protein’s ability to interact with microtubules and increase its tendency to form abnormal filaments (Brackhan et al., 2023; Parra Bravo et al., 2024). In contrast, some tauopathies, like AD, involve TAU protein dysfunction due to other modifications, such as excessive phosphorylation. This leads to the formation of neurofibrillary tangles that damage neurons. Regardless of the cause of TAU disruption, tauopathies typically involve changes in synapse function, neuronal death, abnormal protein accumulation, and brain inflammation (Lee et al., 2001; Devi, 2023). Cell death is now understood to be a key factor propelling the progression of inflammation. Growing research suggests that an intricate and close relationship exists between inflammatory processes and TAU protein, creating a vicious circle generated by activated microglia and damaged neurons (Nilson et al., 2017; Langworth-Green et al., 2023). However, despite extensive research efforts, an effective treatment for tauopathies remains elusive. Consequently, the development of neuroprotective therapies capable of breaking this vicious cycle and thereby mitigating the neurodegenerative process has become a key research priority.

The inflammatory cytokine TNF (tumor necrosis factor) plays a pivotal role in coordinating the inflammatory immune response (van Loo and Bertrand, 2023). RIPK1 (Receptor-Interacting Protein Kinase 1) has been identified as a critical early-stage controller of TNF receptor signaling, orchestrating both inflammatory response mechanisms and the activation of various cell death mechanisms, particularly apoptosis and necroptosis. RIPK1 is a death-domain containing Ser/Thr kinase with several functions in different cellular pathways (Degterev et al., 2019; Yuan et al., 2019; Mifflin et al., 2020; Li and Yuan, 2023). Its structural scaffold supports the canonical NF-κB pathway, while its enzymatic activity not only triggers different forms of cell death (necroptosis and apoptosis) but also amplifies inflammation by stimulating the production of inflammatory signaling molecules (Li and Yuan, 2023). In pathological states, upregulation of death receptor family ligands, such as TNF, can sensitize cells in the central nervous system (CNS) to apoptosis and necroptosis, that is mediated by RIPK1(Yuan et al., 2019). RIPK1’s role in regulating necroptosis is both complex and critical. Its effects range from initiating to inhibiting cell death processes, with its specific functions dependent on the cellular context and cell type involved (Zhang et al., 2017). Related to AD, previous studies have shown that RIPK1 is increased in patient brains, specifically in microglial cells surrounding *β*-amyloid plaques, indicating that it might be involved in regulating disease-associated microglial function (Ofengeim et al., 2017). However, the implication of RIPK1 associated with TAU or TAU neurofibrillary tangles is unknown, although it is well known that glial contribution is critical for tauopathies. There is only one study where it has been reported that TAU hyperphosphorylation is associated with the necroptosis process both *in vitro* (HT22 cells) and *in vivo* (P301S transgenic mice), and that RIPK1 inhibition by Nec-1 has a protective effect (Dong et al., 2022). Because TAU is a protein involved in multiple neurodegenerative diseases, such as FTD and PSP among others, in this study we addressed the possible implication of RIPK1 in tauopathies and the therapeutic potential of the specific inhibitor GSK2982772, which is currently being tested in clinical trials for inflammatory diseases (Tompson et al., 2021a; Tompson et al., 2021b; Weisel et al., 2021; Ludbrook et al., 2024), as a new therapeutic approach for tauopathies.

## 2 Methods

### 2.1 Animals and stereotaxic injections

**Figure 1:**
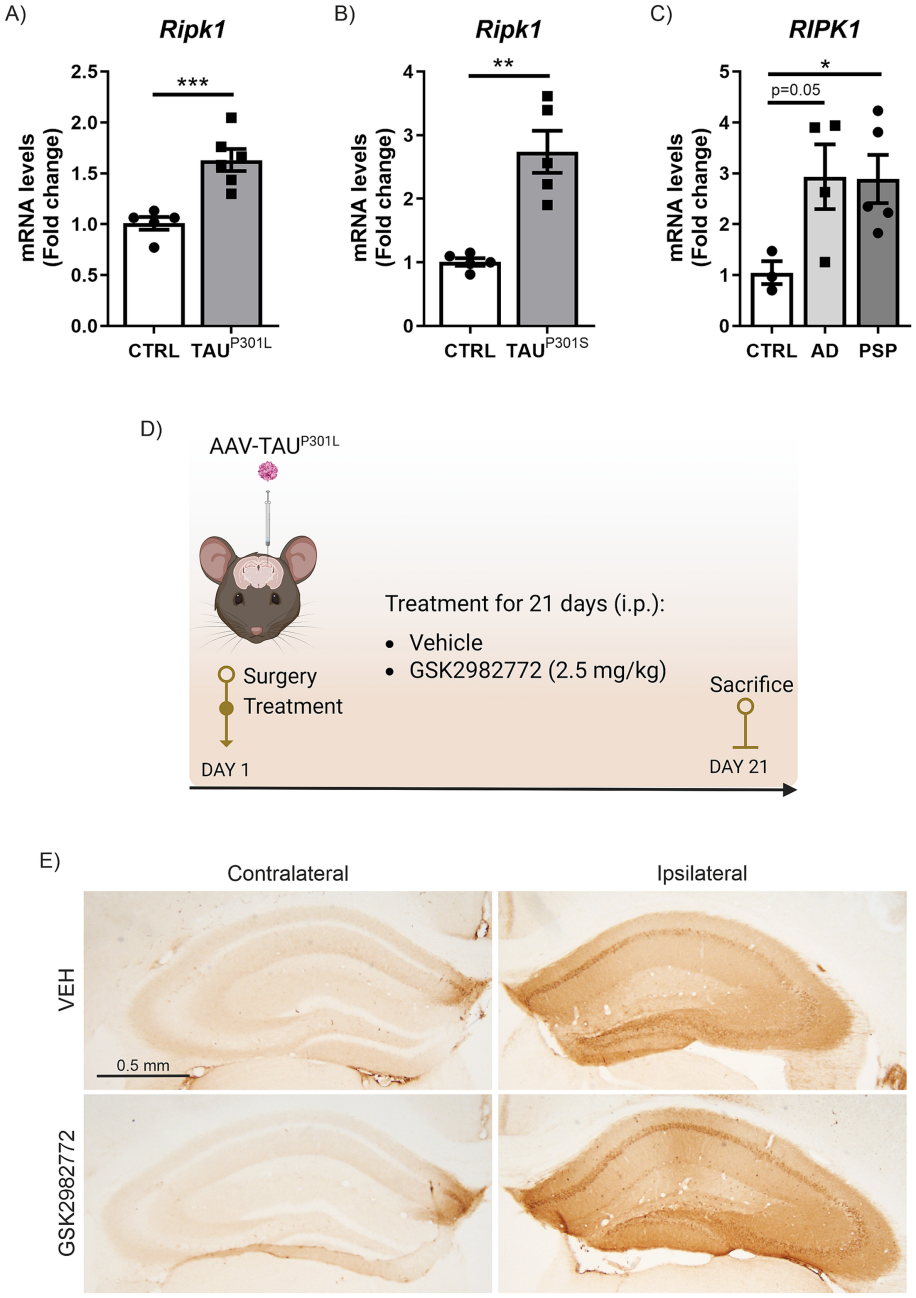
Increased *RIPK1* mRNA expression in tauopathies. qRT-PCR determination of mRNA levels of *Ripk1* in hippocampus of (**A**) AAV-TAU^P301L^ mouse model, (**B**) transgenic TAU^P301L^ mice and (**C**) post-mortem AD and PSP patient samples; *n* = 3–6 samples ± SEM. The asterisks represent the difference in significance: *p < 0.05, **p < 0.01, ***p < 0.001; comparing each group with the control condition according to t-Student test. (**D**) Timeline representation of the experimental design: day 1, we performed the surgery and started the treatment with VEH or GSK2982772 (2.5 mg/kg, i.p.); and day 21, we sacrificed animals, and brain tissue were perfused for immunohistochemistry or immunofluorescence staining, or dissected for qRT-PCR analysis. (**E**) Representative images of the immunohistochemistry against human TAU in 30 μm-thick sections of contralateral and ipsilateral hippocampus from each experimental group.

12-month-old transgenic mice overexpressing hTAU^P301S^ protein (B6;C3-Tg(Prnp-MAPT*P301S)PS19Vle/J, The Jackson Laboratory) were used. The controls animals are wildtype littermates of the same sex and age. Each experimental group comprised 4–5 mice. Regarding the adeno-associated viral vector mouse model, each experimental group consisted of *n* = 4–5 male wild type C57BL/6 mice of 6 month of age. The mice were bred and housed (three to four mice per cage) in temperature-controlled cages (23°C) under a 12/12 h light/dark cycle with free access to water and standard chow in the Autonomous University of Madrid Animal Core. Viral vector injections were performed under ketamine/xylazine anesthesia (8 mg/kg ketamine and 1.2 mg/kg xylazine) on adult mice. Surgery was performed using a stereotaxic frame (RWD, Sugar Land, USA) and a 5 μL Hamilton syringe fitted with a pulled glass capillary tube (outer diameter of 60–80 μm). Recombinant adeno-associated viral vectors of serotype 9, which express the human protein TAU^P301L^ under control of the human synapsin 1 gene promoter (AAV-TAU^P301L^), were injected in the right hippocampus (ipsilateral side) as described elsewhere (Lastres-Becker et al., 2014; Cuadrado et al., 2018; Castro-Sánchez et al., 2020). The overexpressed isoform is microtubule-associated protein TAU isoform 2 (NP_005901), full-length (FL) 4R isoform (2N4R) (Rizzu et al., 2000; Brackhan et al., 2023). Briefly, 2 μL of AAV suspension containing 3.5 × 10E12 pv/ml were injected at the stereotaxic coordinates −2.00 mm posterior, −1.5 mm lateral, and − 1.8 mm ventral relative to bregma, and the left hippocampus (contralateral side) was used as a control. Animals were treated intraperitoneal (i.p.) with vehicle (VEH) (5% N-metil-2-pirrolidona, 5% solutol HS-15 and 95% saline) and GSK2982772 (2.5 mg/kg) final volume 150 μL, 4 h later from the stereotaxic injection of AAV-hTAU^P301L^. This compound or the vehicle were given daily (i.p.) during 3 weeks (Figure 1D). All experiments were performed in a P2 biosafety facility and by certified researchers according to regional, national, and European regulations concerning animal welfare and animal experimentation, and were authorized by the Ethics Committee for Research of the Universidad Autónoma de Madrid with Ref PROEX 130.4/21. Every effort was taken to minimize the number of animals used and their suffering. The total number of mice used in the GSK2982772 study was 11 (*n* = 11 WT-VEH; *n* = 11 WT- GSK2982772).

### 2.2 Randomization and blinding

Animals were randomized for treatment. Data collection and evaluation of all experiments were performed blindly of the group identity. The data and statistical analysis with the recommendations on experimental design and analysis in pharmacology (Curtis et al., 2018).

### 2.3 Analysis of mRNA levels by quantitative real time PCR

Total RNA extraction, reverse transcription, and analysis of mRNA levels by quantitative real time PCR (qRT-PCR) was performed as described in a previous article (Silva-Llanes et al., 2023). One microgram of RNA from each sample was treated with DNase (Invitrogen) and reverse-transcribed using high capacity RNA-to-cDNA Master Mix (Applied Biosystem). Primer sequences are presented in Supplementary Table 1A. Data analysis was based on the 2-ΔΔCT method with normalization of the raw data by the geometric mean of the housekeeping genes *Actb*, *Gapdh* and *Tbp* (Applied Biosystems). All PCRs were performed in triplicate.

### 2.4 Immunohistochemistry on mouse tissues

Mouse brain tissue was sectioned at 30 μm on a cryostat (Leica CM 1950) and stained as free-floating sections with Netwell baskets. To confirm that the stereotaxic delivery was successful slices from the hippocampus region of the brain were stained with an antibody that specifically recognizes the human TAU protein. As previously described (Lastres-Becker et al., 2014; Cuadrado et al., 2018; Castro-Sánchez et al., 2020), a standard avidin-biotin immunohistochemical protocol was used. Primary and secondary antibodies are described in Supplementary Table 1B. Briefly, the primary antibody used was anti-human TAU (TAU (HT-7)), for 24 h at RT. After washing, the sections were first incubated with the secondary biotinylated antibody and then with the ABC kit system to increase sensitivity, and developed using 3, 3 -diaminobenzidine (DAB). Finally, tissues dehydrated in ethanol, and cleared in xylene. Sections were mounted with DEPEX and coverslipped. The immunohistochemistry images were captured using a directed Axiophot microscope (Zeiss) with transmitted light and epifluorescence. It has a camera with DP70 color with a DP Controller image capture system. Mice that did not show correct hTAU expression were discarded for further analysis (Figure 1E).

### 2.5 Immunofluorescence on mouse tissues

Immunofluorescence assays were performed on 30-μm thick coronal brain sections. The protocol followed was previously described (Galán-Ganga et al., 2021). Primary and secondary antibodies are described in Supplementary Table 1B. Images were taken at spectral confocal microscopes Zeiss LSM710 (SEMOC, Instituto de Investigaciones Biomédicas “Sols-Morreale”) or Leica TCS SP5 (SIdI, School of Medicine, Universidad Autónoma de Madrid). A total of 2–3 hippocampal sections *per* side and condition were analysed as follows. The area of the dentate gyrus (ipsilateral vs. contralateral) from mice treated with vehicle or GSK2982772 stained with DAPI and the CALBINDIN-D28K fluorescence intensity was analysed by the Image J 1.54d. The images were transformed into 16-bit and with the “Free Hand Selection” tool, we manually selected only the dentate gyrus area of each image stained with DAPI. The dimension of the dentate gyrus or CALBINDIN-D28K fluorescence intensity inside the selected area was quantified using the “Measure” tool in Image J program and the raw results measured in inches were represented. (*n* = 3–4 animals per experimental group).

### 2.6 Stereological analysis of microgliosis and astrogliosis

Cell counts were performed every eight sections (30 μm-thick) using Fiji Software.^1^ For each mouse, 2–3 hippocampal sections were used for analysis (Schindelin et al., 2012). The error coefficient attributable to the sampling was calculated according to Gundersen and Jensen (1987), and values ≤0.10 were accepted (*n* = 3–4 animals per experimental group).

### 2.7 Human samples

Samples and data from patients included in this study were provided by the Biobank Banco de Tejidos CIEN (PT17/0015/0014), integrated in the Spanish National Biobanks Network and they were processed following standard operating procedures with the appropriate approval of the Ethics and Scientific Committees. Immediately after brain extraction, midsagittal sectioning was performed to separate the right and left hemispheres of the brain. The left hemisphere was fixed in 10% buffered formalin for at least 3 weeks, and the hemisphere right were sliced and these slices were quick frozen fresh at −50°C (in NOVEC) and were immediately placed in at −80°C, where they were stored. The frozen postmortem hippocampal tissues were obtained from four control, four AD patients and five progressive supranuclear palsy (PSP) patients (Supplementary Table 1C), within less than 6 h postmortem interval, according to the standardized procedures of Banco de Tejidos de la Fundación CIEN (Madrid, Spain). These frozen samples were used for RNA and qRT-PCR analysis. The protocol used was similar to the one described in (Galán-Ganga et al., 2021).

### 2.8 Statistical analysis

Data are presented as mean ± SEM (Standard Error of the Mean). To confirm which statistical test had to be used, we employed GraphPad InStat 3 including the analysis of the data to normal distribution via the Kolmogorov–Smirnov test. Furthermore, statistical assessments of differences between groups were analysed (GraphPad Prism 5, San Diego, CA). Unpaired Student’s t-tests or two-way ANOVA with *post hoc* Bonferroni were used as appropriate.

### 2.9 Data availability

The data that supports the findings of this study is available by contacting the corresponding author.

## 3 Results

### 3.1 TAU-induced upregulation of *RIPK1* mRNA expression in murine tauopathy models and individuals diagnosed with AD and PSP

We aimed to determine the mRNA expression levels of RIPK1 in two murine tauopathy models (AAV-TAU^P301L^ and Tg- TAU^P301S^) and compare them with what occurs in humans with primary (PSP) and secondary (AD) tauopathies, to establish whether abnormal TAU protein is linked to alterations in RIPK1 across both animal models and human tauopathy patients. First, we analyzed hippocampal samples from a tauopathy model consisting of mice stereotaxically injected into the right hippocampus with an AAV-hTAU^P301L^ vector for 21 days (Lastres-Becker et al., 2014; Cuadrado et al., 2018; Castro-Sánchez et al., 2020; Galán-Ganga et al., 2021), which mimics the earlier stages of the pathology. Analysis of the samples by qRT-PCR showed that overexpression of hTAU^P301L^ induced a highly significant increase in *Ripk1* mRNA levels (Figure 1A). The increase in *Ripk1* mRNA expression levels was confirmed in the hippocampus of transgenic mice overexpressing the hTAU^P301S^ protein (Tg- TAU^P301S^) at 12 months of age, which mimics the later stages of the pathology (Figure 1B). Finally, to assess the relevance of RIPK1 in human TAU pathology, we analyzed post-mortem hippocampal biopsies from four asymptomatic control donors, four AD patients with neurofibrillary tangles (a secondary tauopathy) (Lastres-Becker et al., 2014), and five patients with PSP, a 4R-tauopathy. In Figure 1C can be observed that *RIPK1* mRNA levels were elevated in the samples from AD patients, approaching significance (*p* = 0.05), and reached statistically significant levels in samples from PSP patients. Taken together, our data indicate an increase in *RIPK1* mRNA expression in both murine models and human samples of primary and secondary TAU-related pathologies.

### 3.2 Treatment with GSK2982772 reduces the astrogliosis linked to TAU^P301L^ overexpression

**Figure 2:**
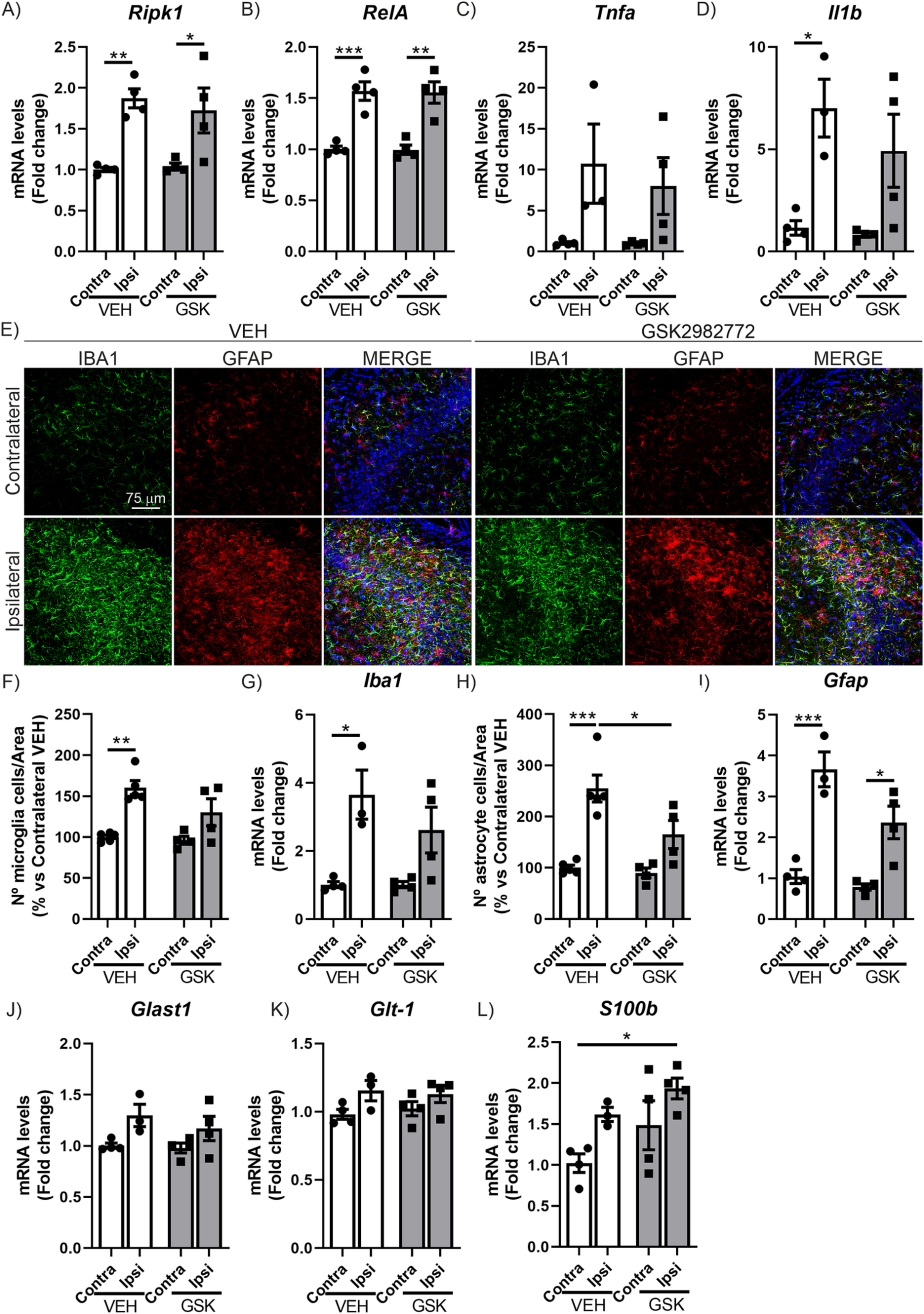
GSK2982772 treatment modulates hippocampal astrogliosis induced by TAU^P301L^ overexpression. qRT-PCR determination of mRNA levels of **(A)** *Ripk1*, **(B)** *RelA*, **(C)** *Tnfa* and **(D)** *Il1b* genes at the hippocampus of mice treated with VEH or GSK2982772, *n* = 3–4 samples ±SEM. **(E)** Immunofluorescence of IBA1 (green) and GFAP (red), microglial and astrocytic markers, respectively, of 30 μm-thick sections in the CA3 region of hippocampus of mice treated with VEH or GSK2982772, n = 3–4 samples ± SEM. **(F)** Quantification of number of IBA1^+^ microglial cells, **(G)** qRT-PCR determination of mRNA levels of *Iba1* gene, **(H)** quantification of number of GFAP^+^ astrocytic cells and **(I)** qRT-PCR determination of mRNA levels of *Gfap* gene at the hippocampus of mice treated with VEH or GSK2982772, *n* = 3–4 samples ±SEM. qRT-PCR determination of mRNA levels of **(J)** *Glast1*, **(K)** *Glt-1*, and **(L)** *S100b* genes at the hippocampus of mice treated with VEH or GSK2982772, *n* = 3–4 samples ±SEM. Differences in significance are symbolized by asterisks: *p < 0.05, **p < 0.01, ***p < 0.001, comparing each group with the contralateral hippocampi from Contra-VEH mice or the indicated groups, according to two-way ANOVA followed by Bonferroni post-test.

It has been reported that inhibition of RIPK1 kinase activity has shown efficacy in a wide range of animal models of human diseases related to inflammation (Polykratis et al., 2014; Chan et al., 2019; Degterev et al., 2019; Mifflin et al., 2020; Patel et al., 2020; Wang et al., 2021; Kim et al., 2023; Li and Yuan, 2023). Among RIPK1 inhibitors, GSK2982772 binds to RIPK1 with high kinase specificity and effectively inhibits TNF-induced necroptosis (Harris et al., 2017; Khoury et al., 2020). Additionally, GSK2982772 has progressed to phase IIa clinical trials for the treatment of non-neurological disorders (Weisel et al., 2017; Tompson et al., 2021a; Weisel et al., 2021; Ludbrook et al., 2024). Among all available RIPK1 inhibitors, we chose GSK2982772 because its high potency that may predict a low oral dose in humans (Harris et al., 2017). However, GSK2982772 brain penetrance is compromised in physiological conditions, mainly due to the effect of efflux transport of P-glycoprotein (Harris et al., 2017). This pharmacokinetic limitation has hindered the use of this inhibitor to test its potential therapeutic effect in central nervous system diseases. Nevertheless, several studies indicate that blood–brain barrier (BBB) permeability may be increased in patients with various neurodegenerative diseases as well as in different animal models (Knox et al., 2022; Bruno et al., 2024). This may be due to, first, a reduction in the activity of efflux pumps in the BBB epithelium (Deo et al., 2014) and, second, to a reduction in BBB integrity, which often correlates with levels of neuroinflammation (Preis et al., 2024). In line with the above, the use of the AAV-TAU^P301L^ model could be a good opportunity to test the therapeutic potential of this drug candidate in the CNS. Therefore, in this study we determined if treatment with this RIPK1 inhibitor could have anti-inflammatory and/or neuroprotective effects, in the model of AAV-TAU^P301L^ overexpression (Figure 1D). Initially, we examined the brain inflammation mechanism linked to this AAV-TAU^P301L^ model by assessing the gene expression of *Ripk1* and components of the inflammatory NF-κB signaling cascade (*RelA*, *Tnfa*, and *Il1b*). The results depicted in Figures 2A–D indicate that administering GSK2982772 does not modify the expression patterns of these inflammation-promoting genes, which are upregulated due to TAU^P301L^ overexpression. In addition to the increased expression of pro-inflammatory molecules, another characteristic of tauopathies is the presence of reactive astrogliosis and microgliosis. Therefore, we next aimed to determine whether treatment with GSK2982772 influenced the gliosis associated with TAU^P301L^ overexpression by analyzing the expression of IBA1 (ionized calcium-binding adapter molecule 1) and GFAP (Glial Fibrillary Acidic Protein), specific markers of microgliosis and astrogliosis, respectively, using stereology and qRT-PCR. As shown in Figure 2E, TAU^P301L^ overexpression induces a significant increase in reactive microgliosis and astrogliosis. Stereological analysis of microgliosis (Figure 2F) indicates that GSK2982772 treatment reduces the number of these cells (19%), which is corroborated by the results obtained through IBA1 mRNA level analysis (28.5%) (Figure 2G), although this decrease does not reach statistical significance. Similarly, we analyzed whether GSK2982772 treatment could modulate astrogliosis. GSK2982772 treatment significantly reduced TAU-induced astrogliosis (35%), both in terms of astrocyte numbers (Figure 2H) and *GFAP* mRNA levels (Figure 2I). No changes were observed in the morphology of reactive microglia or astroglia. This difference in response between microgliosis and astrogliosis led us to further analyze the expression of astrocytic markers such as *Glast-1*, *S100b*, and *Glt-1*. It has been described that astrocytes are essential for regulating extracellular glutamate levels in the synaptic cleft, helping to reduce the risk of excitotoxicity in neurons, by glutamate aspartate transporter 1 (GLAST-1) and glutamate transporter-1 (GLT-1) (Rose et al., 2017; Mahmoud et al., 2019). Our results indicate that TAU^P301L^ overexpression slightly induces the expression of *Glast1* and *Glt-1*, which are not affected by treatment with the RIPK1 inhibitor (Figures 2J,K). Additionally, we analyzed the expression levels of *S100b*, a marker abundantly expressed in astrocytes, which is involved in regulating calcium homeostasis by modulating calcium-dependent processes that are critical for synaptic plasticity (Donato et al., 2013). Our data indicate that TAU^P301L^ overexpression increases the expression of *S100b*, which is further exacerbated by treatment with GSK2982772 (Figure 2L), supporting a protective role in relation to the TAU protein, as has been previously suggested (Moreira et al., 2021). Overall, our data suggest that GSK2982772 treatment predominantly affects the astrogliosis linked to TAU overexpression.

### 3.3 Hippocampal neurodegeneration induced by TAU^P301L^ overexpression is not altered following treatment with GSK2982772

**Figure 3:**
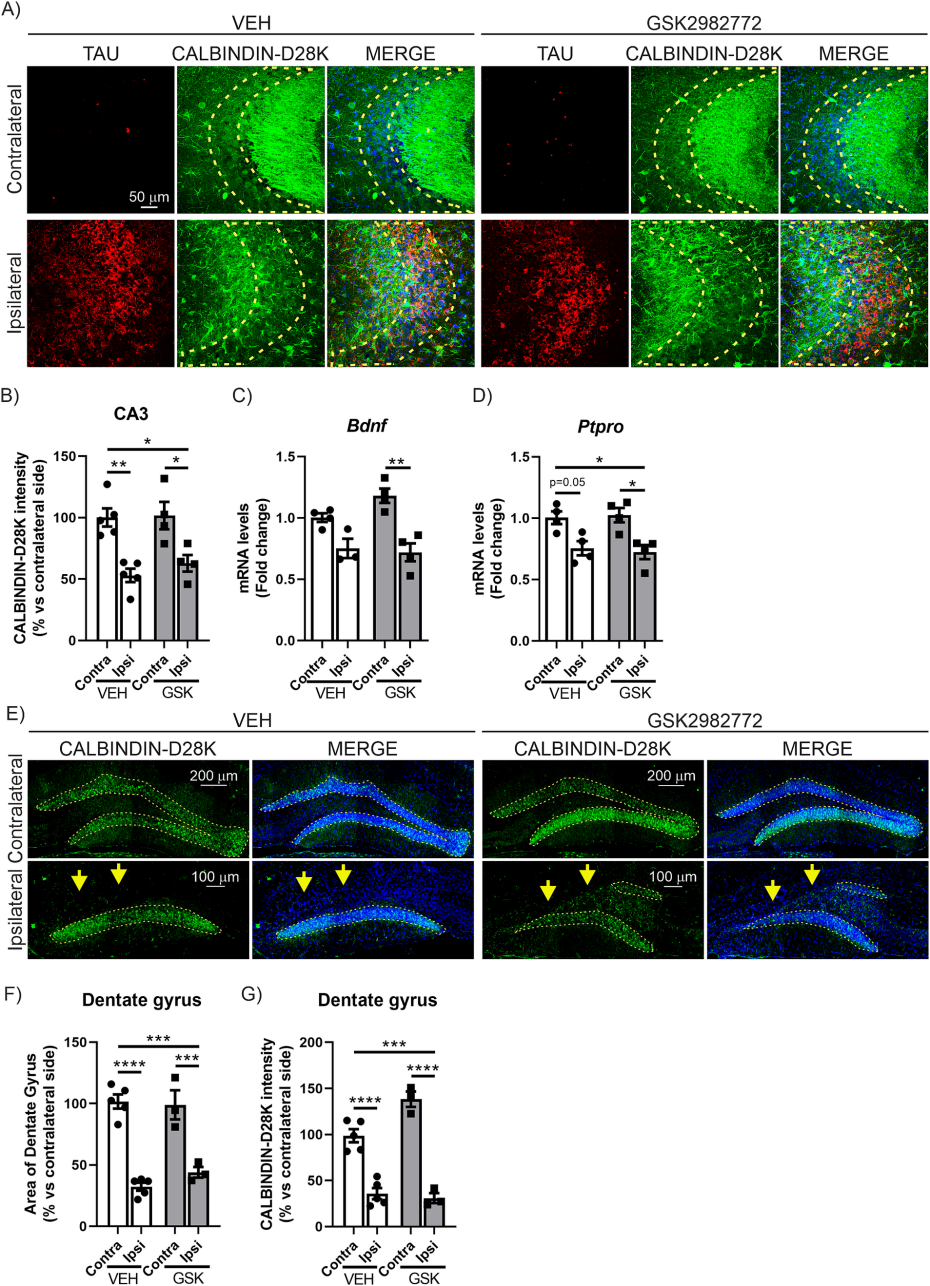
GSK2982772 does not exhibit a neuroprotective effect in the CA3 or granular cell layer of the dentate gyrus in the hippocampus caused by TAU^P301L^ overexpression. **(A)** Immunofluorescence of human TAU (red) and CALBINDIN-D28K (green), a synaptic plasticity marker, of 30 μm-thick sections in the CA3 region of hippocampus of mice treated with VEH or GSK2982772, *n* = 3–4 samples ± SEM. Regions of interest (ROI) are outlined in yellow. Quantification of **(B)** CALBINDIN-28 K fluorescence intensity in ROI at the hippocampus of mice treated with VEH or GSK2982772, n = 3–4 samples ±SEM. qRT-PCR determination of mRNA levels of **(C)** *Bdnf* and **(D)** *Ptpro* genes at the hippocampus of mice treated with VEH or GSK2982772, *n* = 3–4 samples ±SEM. **(E)** Immunofluorescence of CALBINDIN-D28K, of 30 μm-thick sections in the dentate gyrus of hippocampus of mice treated with VEH or GSK2982772, *n* = 3–4 samples ± SEM. Regions of interest (ROI) are outlined in yellow. Quantification of **(F)** the area of the dentate gyrus, and **(G)** CALBINDIN-D28K fluorescence intensity in ROI at the hippocampus of mice treated with VEH or GSK2982772, *n* = 3–4 samples ±SEM. The asterisks describe the difference in significance: *p < 0.05, **p < 0.01, ***p < 0.001, ****p < 0.0001, comparing each group with the contralateral hippocampi from Contra-VEH mice or the indicated groups, according to two-way ANOVA followed by Bonferroni post-test.

Previous studies in our laboratory show that the overexpression of TAU^P301L^ causes severe neurodegeneration in the dentate gyrus (DG) and CA3 regions at the hippocampus (Cuadrado et al., 2018; Castro-Sánchez et al., 2020; Galán-Ganga et al., 2021). Therefore, our next step focused on analyzing the effect of RIPK1 inhibition on the neurodegenerative process caused by TAU^P301L^ overexpression at these regions. The analysis of CALBINDIN-D28K levels in CA3, via immunofluorescence techniques reaffirmed our earlier findings in this model (Cuadrado et al., 2018; Galán-Ganga et al., 2021). This crucial calcium-binding protein, which serves a buffering function, exhibits an inverse relationship with TAU^P301L^ overexpression levels. The results clearly demonstrated that elevated TAU^P301L^ overexpression leads to a marked reduction in CALBINDIN-D28K levels (Figures 3A,B). However, treatment with GSK2982772 was unable to restore CALBINDIN-D28K levels, as shown in Figures 3A,B, suggesting that RIPK1 inhibition does not reverse the neurodegeneration caused by TAU^P301L^ overexpression. To further support these findings, we analyzed the mRNA levels of Brain derived neurotrophic factor (*Bdnf*) and protein tyrosine phosphatase receptor type O (*Ptpro*), two markers of synaptic plasticity. In mature neural tissue, BDNF primarily serves to modulate synaptic connections (Lu et al., 2014). Meanwhile, PTPRO functions as an adhesion molecule at synapses, facilitating the establishment of new synaptic junctions (Jiang et al., 2017). Our results suggest that TAU^P301L^ overexpression reduces the mRNA expression levels of *Bdnf* (Figure 3C) and *Ptpro* (Figure 3D), and that treatment with GSK2982772 does not lead to any improvement in these synaptic plasticity markers in the CA3 region. In addition to the CA3 region, we also analyzed the DG area in a similar manner. Fluorescent labeling of the DG region using DAPI, which selectively attaches to AT-rich DNA segments, revealed that TAU^P301L^ overexpression led to partial deterioration of the granule cell layer on the ipsilateral side in mice given VEH (Figures 3E,F). However, this deterioration was not modified by administered GSK2982772. To validate this finding, we examined the expression levels of CALBINDIN-D28K. As shown in Figures 3E–G, TAU^P301L^ overexpression results in reduced CALBINDIN-D28K levels in the DG of VEH-treated mice compared to the opposite hemisphere. Moreover, GSK2982772 treatment did not exhibited any modifications in CALBINDIN-D28K expression, supporting the results obtained from DAPI staining. Taken together, these data suggest that treatment with the RIPK1 inhibitor does not have a neuroprotective effect against TAU^P301L^ overexpression in the hippocampus.

## 4 Discussion

At present, there are no treatments available that can decelerate or stop the progression of neurodegeneration in disorders linked to TAU protein anomalies, collectively termed tauopathies. A shared characteristic among these conditions is the persistent and sustained activation of neuroinflammation, which intensifies the deterioration of neurons. Consequently, there is a pressing need to uncover the underlying pathways involved in these processes and evaluate their viability as targets for therapy. In this regard, RIPK1 has emerged as a promising candidate for therapeutic intervention. In this work, we have demonstrated for the first time that it is a commonly altered mechanism in various murine models of tauopathy (AAV-TAU^P301L^ and Tg-TAU^P301S^), as well as in samples from patients with primary tauopathy, such as PSP, and secondary tauopathy, such as AD. The encouraging findings from our initial research prompted us to investigate the potential anti-inflammatory and/or neuroprotective properties of a RIPK1 inhibitor called GSK2982772 in our AAV- TAU^P301L^ -based tauopathy model. Despite our expectations, the administration of GSK2982772 yielded only improvements in reducing astrocyte activation, with no impact on neurodegeneration.

The data obtained from two different murine models of tauopathy (Figure 1) clearly indicate that the pathological process associated with TAU leads to an increase in *RIPK1* mRNA expression levels. These results are consistent with the study by Dong et al. (2022), which demonstrates that Tg-TAU^P301S^ mice exhibit an age-dependent increase in RIPK1 levels, confirming that pTAU activates neuronal necroptosis. It is interesting to note that we found similar results of increased *RIPK1* mRNA expression levels in samples from both PSP and AD patients (Figure 1). Although a previous study had already reported elevated RIPK1 levels in AD patients (Ofengeim et al., 2017), since this disease is a secondary tauopathy underlying APP amyloidosis, it could not be confirmed that this increase in RIPK1 levels was a direct consequence of TAU hyperphosphorylation and aggregation. However, the fact that PSP patients, a neuroglial 4R tauopathy (Kovacs et al., 2020), also exhibit elevated *RIPK1* levels suggests a more direct role of TAU involvement. Our data provide new insight into the mechanism by which the TAU protein induces a common RIPK1 pathway in different tauopathies.

The observation of an increase in RIPK1 levels in these tauopathies led us to explore whether its modulation could serve as a therapeutic target to first inhibit neuroinflammation and subsequently slow neurodegeneration. Due to the growing interest in this protein, various types of RIPK1 inhibitors have been developed with potential therapeutic applications (Martens et al., 2020), with the GSK2982772 compound currently showing the best results in the treatment of inflammatory diseases (Harris et al., 2017; Weisel et al., 2017; Tompson et al., 2021a; Tompson et al., 2021b; Weisel et al., 2021; Ludbrook et al., 2024). Nec-1 and Nec-1 s have been utilized in various experimental setups modeling diseases associated with necroptosis, showing potential for therapeutic applications. However, these compounds display limited effectiveness and suboptimal pharmacokinetic characteristics and its use is restricted due to its metabolic instability and unintended off-target effects (Teng et al., 2005; Degterev et al., 2013; Cao and Mu, 2021). Therefore, in our study, we administered GSK2982772 treatment for 21 days in our AAV-TAU^P301L^ tauopathy model. Our findings indicate that blocking RIPK1 activity leads to a decrease in the reactive astrocyte response triggered by excessive TAU^P301L^ protein (Figure 2). We also noticed a small reduction in microglial activation. The RIPK1 inhibitor treatment did not appear to influence the levels of inflammatory signaling molecules or alter the progression of neuronal damage in the hippocampus caused by TAU^P301L^ overexpression (Figures 2, 3). This lack of effectiveness of GSK2982772 treatment in the degenerative profile of our tauopathy model may suggest, firstly, that although necroptosis is present, other mechanisms such as inflammosome/pyroptosis may be more predominant in the TAU-associated neuroinflammation/neurodegeneration. In fact, it has been reported that TAU can modulate the neuroinflammatory process through NLRP3 inflammasome activation (Ising et al., 2019; Stancu et al., 2019; Lemprière, 2020; Stancu et al., 2022). Recent research has revealed that TAU protein forms a complex with polyglutamine binding protein 1 (PQBP1) *in vitro* (Jin et al., 2021). This interaction appears to trigger the innate immune response in primary microglial cells, but in a PQBP1-dependent manner. Furthermore, the presence of PQBP1 in microglia seems to play a crucial role in the inflammatory response caused by TAU protein in brain tissue. So, it seems that TAU is capable of triggering various inflammation-related signaling cascades.

Lastly, one additional approach would be to test higher doses of the GSK2982772 compound in future experiments to potentially amplify the observed effects on astrogliosis and microgliosis. Studies have shown that GSK2982772 exhibits limited brain concentration by the efflux transporter protein known as P-gp (Harris et al., 2017). The main issue with RIPK1 inhibitors is their ability to cross the BBB. Many of these inhibitors are unable to cross it, and those that do often exhibit high liver toxicity deemed unrelated to RIPK1 inhibition, making them unsuitable for the treatment of neurodegenerative diseases, where long-term chronic treatment is required (Grievink et al., 2020).

Overall, our results suggest that modulation of RIPK1 by the small molecule GSK2982772 decrease the astrocytosis associated with TAU-induced degeneration but, mainly due by the complexity of the multiple cascades involved in the neuroinflammatory process, beneficial effect directly on neurodegeneration is not observed.

## Data Availability

The original contributions presented in the study are included in the article/Supplementary material, further inquiries can be directed to the corresponding author.

## Footnotes

^1^ http://fiji.sc/Fiji
