# Supplementary material for: RIPK1 expression and inhibition in tauopathies: implications for neuroinflammation and neuroprotection
Source: Front Neurosci. 2025 Jan 27;18:1530809. doi: 10.3389/fnins.2024.1530809 (PMC11808139; doi:10.3389/fnins.2024.1530809)
Supplement: Supplementary file 1 [file Table_1.docx]

**Supplementary Table 1A:** List of primers used in this study.

| **Gene Product** | **Forward Primer** | **Reverse Primer** |
| --- | --- | --- |
| *Actb* | 5’ TCCTTCCTGGGCATGGAG 3’ | 5’ AGGAGGAGCAATGATCTTGATCTT 3’ |
| *Bdnf* | 5’ GATGCCGCAAACATGTCTATGA 3’ | 5’ TAATACTGTCACACACGCTCAGCTC 3’ |
| *Gapdh* | 5’ CGACTTCAACAGCAACTCCCACTCTTCC 3’ | 5’ TGGGTGGTCCAGGGTTTCTTACTCCTT 3’ |
| *Gfap* | 5’ TCCTGGAACAGCAAAACAAG 3’ | 5’ CAGCCTCAGGTTGGTTTCAT 3’ |
| *Iba1* | 5’ GTCCTTGAAGCGAATGCTGG 3’ | 5’ CATTCTCAAGATGGCAGATC 3’ |
| *Il1b* | 5’ CTGGTGTGTGACGTTCCCATTA 3’ | 5’ CCGACAGCACGAGGCTTT 3’ |
| *Ptpro* | 5’ TTGTAAGCGCCACGGAGAA 3’ | 5’ TTGTTGTCATCTTGCACAGCC 3’ |
| *Rela* | 5’ GGCCTCATCCACATGAACTT 3’ | 5’ CACTGTCACCTGGAAGCAGA 3’ |
| *Ripk1* | 5’ GACTTCCAGACACCAAGCCA 3’ | 5’ ACTGCCTTCCCAGGTTTTCC 3’ |
| *Tbp* | 5’ TGCACAGGAGCCAAGAGTGAA 3’ | 5’ CACATCACAGCTCCCCACCA 3’ |
| *Tnfa* | 5’ CATCTTCTCAAAATTCGAGTGACAA 3’ | 5’ TGGGAGTAGACAAGGTACAACCC 3’ |

**Supplementary Table 1B:** List of antibodies used in this study.

| Antibody | Source | Catalog Number | Dilution |
| --- | --- | --- | --- |
| Alexa Fluor 488 donkey anti-mouse IgG | Thermo Fisher Scientific | A21202 | 1:500 IF |
| Alexa Fluor 488 donkey anti-rabbit IgG | Thermo Fisher Scientific | A21206 | 1:500 IF |
| Alexa Fluor 546 donkey anti-mouse IgG | Thermo Fisher Scientific | A31570 | 1:500 IF |
| Alexa Fluor 546 donkey anti-rabbit IgG | Thermo Fisher Scientific | A31572 | 1:500 IF |
| CALBINDIN-D28K | Synaptic Systems | 214.002 | 1:500 IF |
| GFAP | Sigma-Aldrich | G3893 | 1:400 IF |
| IBA1 | Wako Chemicals | 019-19741 | 1:500 IF |

**Supplementary Table 1C:** List of patients used in this study.

| **Code** | **Experimental group** | **Gender** | **Age (years)** |
| --- | --- | --- | --- |
| AD | BCPA 7 | Male | 80 |
| AD | BCPA 143 | Male | 88 |
| AD | BCPA 279 | Female | 98 |
| AD | BCPA 381 | Male | 86 |
| CTRL | BCPA 364 | Male | 43 |
| CTRL | BCPA 587 | Female | 83 |
| CTRL | BCPA 662 | Female | 58 |
| PSP | BCPA 135 | Male | 83 |
| PSP | BCPA 252 | Male | 72 |
| PSP | BCPA 298 | Male | 79 |
| PSP | BCPA 449 | Male | 88 |
| PSP | BCPA 468 | Female | 74 |
